# English and American Infants’ Food

**Published:** 1886-05

**Authors:** 

**Affiliations:** Bonn, Germany, Food Analyst for Rhenish Prussia


### English and American Infants' Food.

By Dr. Stutzer, of Bonn, Germany, Food Analyst for Rhenish Prussia.

The various kinds of artificially Prepared Infants' Food vary much in their composition, and in the proportion of their component parts in regard to digestibility and nutritive value. Inasmuch as the use of artificial Infants' Food has steadily increased, and therefore become of much importance to all classes, the true value of such food should be better known, their manufacture more strictly controlled, and, from time to time, be analyzed and reported through the press.

Large quantities of such foods, which have been manufactured without due consideration requisite for a rational Infants' Food, are annually brought into the market and consumed; whereas, such preparations being deficient in either proper nutrition or disproportionate in their non-nitrogenous and nitrogenous components and inorganic salts, should not be used for the feeding of infants. Such crude kinds of Artificial Food are neither adapted for ready digestion nor for healthy nutrition, and can produce more harm to the Infantile System than, for instance, adulterated wine or spices to an adult.

In order to bring this important matter to a practical issue, I have made an analysis of the more popular English and American Infants' Food, and have arranged the results for ready and comparative reference, in the following table:

| | Nestle's Food. | Carnrick's Soluble Food. | Mellin's Food. | Wells, Richardson & Co.'s Lactated Food. | Horlick's Food. | Dr. Ridge's Patent Food. |
|---|---|---|---|---|---|---|
| Fat | 4.66 | 5.00 | 0.50 | 2.19 | 0.60 | 1.27 |
| Protein substances (albuminoids) | 11.46 | 18.22 | 8.34 | 9.05 | 11.30 | 8.76 |
| Soluble Hydrocarbons (Sugars, Dextrin, etc.) | 41.22 | 26.87 | 60.89 | 25.52 | 65.92 | 1.79 |
| Insoluble Hydrocarbons (Starch, etc) | 35.47 | 40.87 | 18.40 | 52.92 | 13.12 | 78.66 |
| Cellulose | 0.10 | . . . | 0.58 | 1.54 | 0.55 | 0.73 |
| Water | 5.34 | 6.14 | 7.76 | 6.52 | 5.75 | 8.31 |
| Salts and inorganic constituents | 1.75 | 2.99 | 3.53 | 2.26 | 2.76 | 0.48 |
| Amount of nitrogen in protein substances | 1.833 | 2.915 | 1.335 | 1.448 | 1.809 | 1.403 |
| Amount of protein substances readily digestible | 11.09 | 16.45 | 7.38 | 8.35 | 10.85 | 7.97 |
| Proportion of nitrogenous alimentary substances (Protein = 1) | 1:7.7 | 1:4.4 | 1:9.6 | 1:9.2 | 1:7.1 | 1:9.3 |
| The inorganic constituents contain— | | | | | | |
| Lime | 0.390 | 0.645 | 0.155 | 0.390 | 0.060 | 0.060 |
| Phosphoric acid | 0.630 | 0.874 | 0.583 | 0.688 | 0.421 | 0.260 |

The following additional remarks may serve as explanatory to the above:

Nestle's Food.—The amount of protein substance in this food is insufficient, and, consequently, the relative proportion between nitrogenous and the other essential constituents are deficient and imperfect. This proportion is in cow's milk as 1:4, in woman's as 1:6.8. But, as the nitrogenous constituents of Artificial Foods are not so readily and completely digested and assimilated as those of mother's milk, and as protein substances supply the requisite material for the formation of flesh and blood, and, consequently, for the development of infants, it seems to me necessary to increase the relative percentage of nitrogenous substances in proportion to the other component parts, and I would prefer a proportion of one part of the former to 5.0 or 4.5 of the latter, so as to supply the Infantile System with a slightly greater amount of nitrogenous matter. Another important factor in an Infants' Food is the bone-forming material, especially of lime-salts and of phosphates. Nestle's Food is deficient in the former.

Carnrick's Soluble Food is the best of all foods examined and mentioned in the table. It excels the other foods by its greater amount of nitrogenous substances (18.22 per cent.), and by a rationally relative proportion (1:4.4) of its essential constituents. It also contains a larger quantity of the bone-forming inorganic substances and of the solid constituents of milk.

According to the statement of the manufacturer, the milk entering into this food is previously treated with Pancreatine, thus rendering the casein more easy of digestion. This certainly is a very rational method, and, as far as I know, is applied by no other manufacturer of Infants' Food. It is also very pleasant to the taste.

Mellins' Food contains but 0.5 per cent. of fat, and the amounts of bone-forming material and protein substances are deficient. The relative proportion of nitrogenous and non-nitrogenous constituents in this food, namely, 1:9.6, is very objectionable.

Wells, Richardson & Co.'s Lactated Food is deficient in the proportion of its nitrogenous and its non-nitrogenous components, viz., 1:9.2.

Horlick's Food contains but 0.6 per cent. of lime.

Ridge's Patent Food is also of a very deficient composition in its nutritive properties, its relative proportion of nitrogenous to the non-nitrogenous constituents being 1:9.3. The amount of starch is far too great.

The analysis of this limited number of Infants' Foods shows how incomplete and inferior most of them are, and how rarely they meet the requisite conditions of a rational food.—*From Pharmaceut. Central Halle, Berlin*, 1886, *No.* 8, and *Pharmac. Rundschau* (New York), 1886, page 89.

